# Gallium68-Labeled Fibroblast Activation Protein Inhibitor (68Ga-FAPI) PET/CT as an Alternative to Fluoro18-Fluorodeoxyglucose (18F-FDG) PET/CT: Discussion in a Case of Metastatic Adenocarcinoma of Pancreas

**DOI:** 10.7759/cureus.50183

**Published:** 2023-12-08

**Authors:** Abhishek Kumar, Bhola Kumar, Amitabh Kumar Upadhyay, G. S. Muthu, Sujata Mitra

**Affiliations:** 1 Nuclear Medicine, Tata Main Hospital, Jamshedpur, IND; 2 Medical Oncology, Tata Main Hospital, Jamshedpur, IND; 3 Nuclear Medicine, Meherbai Tata Memorial Hospital, Jamshedpur, IND

**Keywords:** fibroblast activation protein inhibitor, 68ga - fapi, 18f - fdg, pet/ct, 18f-fluorodeoxyglucose-positron emission tomography, positron emission tomography, 18f-fluorodeoxyglucose

## Abstract

Positron emission tomography (PET) is an integral part of the imaging of solid tumors in today's oncology practice. The most commonly used PET radiotracer is 18F-Fluorodeoxyglucose (18F-FDG). FDG PET has imaging characteristics of a high tumor-to-background uptake ratio and is used in the detection of primary as well as metastatic sites. However, a significant pitfall is its inability to differentiate between neoplastic and infective lesions. To address this concern, many PET radiotracers have been developed and tried over time, a promising one being radiolabelled fibroblast activation protein inhibitor (FAPI). Fibroblast-activated protein (FAP) is a type II transmembrane glycoprotein expressed by cancer-associated fibroblasts (CAFs); it forms a significant component of the tumor stroma. Since there is over-expression of CAF in the majority of malignancies, it is a potential target for molecular imaging using PET. Several radiolabeled FAP inhibitors have been developed for PET imaging of malignancies and have also been used in theranostic applications.

## Introduction

Molecular imaging has revolutionized the field of imaging. The hybrid imaging modality positron emission tomography/computed tomography (PET/CT) not only gives details on morphology and anatomy but also visualizes the physiological and pathological processes within the organs and lesions at the cellular level. 18F-Fluorodeoxyglucose (18F-FDG) is one of the most widely used PET/CT radiotracers [1]. It is not only used in oncology but also used to detect infective and inflammatory etiology. It also serves as a radiotracer to determine the viability of the left ventricle myocardium in the field of cardiology. Other commonly used PET radiotracers are 68Ga-DOTANOC in cases of neuroendocrine tumors and 68Ga/18F-PSMA in cases of adenocarcinoma prostate [2-5]. These 68Ga radiolabeled PET radiotracers are synthesized at the PET/CT facility from 68Ge/68Ga generators. These portable generators offer the advantage of radiotracer availability at PET/CT centers distant from medical cyclotrons. Unlike in the case of 18F-FDG, these 68Ga-based radiotracers are not dependent on the blood glucose levels of the patient and offer another advantage in performing PET/CT independent of blood glucose levels. The 18F-FDG uptake is dependent on the blood glucose levels of patients and poses a challenge in cases of diabetics, wherein the patient has to be fasting for at least four hours [6]. The time gap between antidiabetic medications and 18F-FDG PET/CT must also be managed so that these medications do not interfere with FDG uptake.

Moreover, FDG uptake is a surrogate for glucose transport/metabolism and is not specific for malignancy [7,8]. There has always been research to develop more specific radiotracers for malignancy. The radiolabeled somatostatin receptor (SSTR) targeting agents and prostate-specific membrane antigen (PSMA) ligands result from translational research. The search for cellular targets led to the discovery of fibroblast activation protein, a transmembrane glycoprotein expressed on activated fibroblasts such as cancer-associated fibroblasts (CAFs) [9]. The new PET radiotracer 68-FAPI targets these CAFs, is unaffected by glucose levels, and offers an advantage over 18F-FDG. It has been used in a variety of solid tumors ranging from hepatobiliary tumors, carcinoma breast, gynecological malignancies, and sarcoma [7,10]. Many radiolabeled fibroblast activation protein inhibitor tracers are being investigated as PET radiotracer agents and for possible use in theranostic applications [11].

## Case presentation

We report a case of biopsy-proven pancreatic adenocarcinoma with liver metastases. The index case is a 61-year-old lady presenting with complaints of nausea, vomiting, and abdominal pain for three months. Ultrasound of the abdomen revealed hepatomegaly with multiple liver lesions suggestive of liver secondaries. Biopsy from the liver lesion suggested metastatic adenocarcinoma. The patient was scheduled for FDG PET/CT for evaluation of these liver lesions and identification of the primary neoplastic site. However, due to uncontrolled diabetes and a fasting blood glucose level of 270 mg/dL, FDG PET/CT was deferred. In consultation with a medical oncologist, 68Ga-FAPI PET/CT was done. Subsequently, after two days, when fasting blood glucose level was under control, 18F-FDG PET/CT was also done to confirm the findings. Both the PET/CT scans were done after obtaining written consent from the patient. The maximum standardized uptake values (SUVmax) were compared for both scans.

68Ga-FAPI PET/CT revealed enhancing primary neoplastic lesion in the head and uncinate process of the pancreas with radiotracer uptake (SUVmax 17.3). Multiple FAPI avid (SUVmax 9.6) liver lesions were also seen, suggesting liver metastases. FAPI avid nodal metastases were noted in portocaval (SUVmax 14.8) and anterior cardiac recess (SUVmax 5.3) regions. FAPI avid deposit (SUVmax 6.0) was also noted in the perinephric region of the right kidney. Background SUVmax of 3.5 was noted on FAPI PET.

18F-FDG PET/CT revealed similar findings in primary and metastatic sites with a primary neoplastic lesion involving the pancreas (SUVmax 7.5) and multiple metastatic liver lesions (SUVmax 6.4). FDG avid nodal metastases in portocaval (SUVmax 5.1) and anterior cardiac recess (SUVmax 1.4) regions with another deposit (SUVmax 2.6) in the perinephric region of the right kidney were noted. These lymph nodes and perinephric deposits were deemed metastatic by two nuclear medicine physicians in view of contrast enhancement and higher uptake values on FAPI PET. Background SUVmax of 2.7 was noted on FDG PET.

On comparison of both these scans, it was noted that both these radiotracers identified the primary and metastatic sites. However, FAPI uptake was higher in primary and metastatic sites, resulting in a better tumor-to-background ratio. Also, FAPI uptake was seen in more number of the liver lesions as compared to FDG, thus identifying a higher burden of tumor volume.

**Figure 1:**
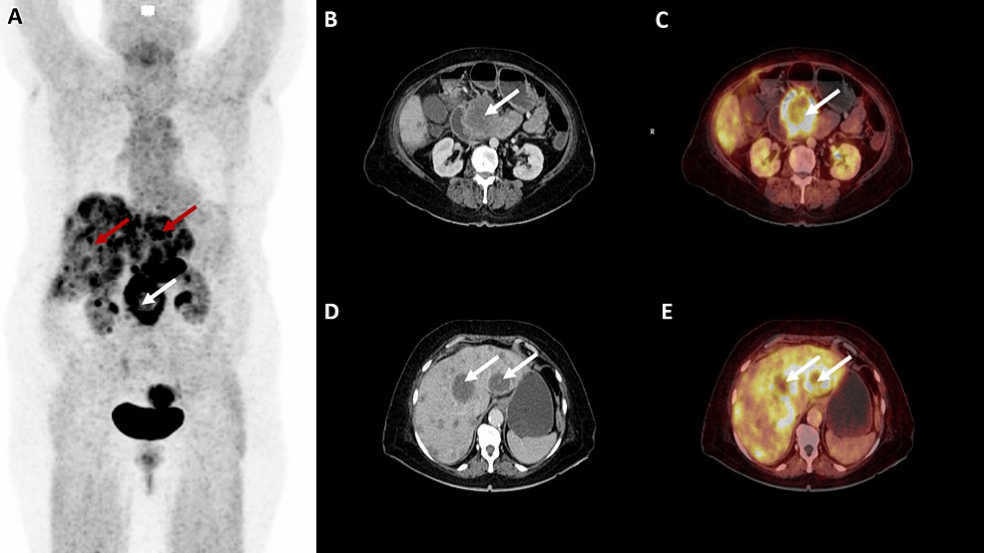
68Ga-FAPI PET/CT images of the patient (A) MIP; (B, D) axial view of CT; (C, E) axial view of 68Ga-FAPI PET/CT fusion. MIP image shows increased FAPI uptake in the region of the pancreas (white arrow) and metastatic liver lesions (red arrows). (B, C) Primary neoplastic lesion involving head and uncinate process of pancreas with FAPI uptake (white arrows). (D, E) Metastatic liver lesions with FAPI uptake (white arrows). MIP: maximal intensity projection; 68Ga-FAPI PET/CT: 68Ga-fibroblast activation protein inhibitor positron emission tomography/computed tomography; FAPI: fibroblast activation protein inhibitor.

**Figure 2:**
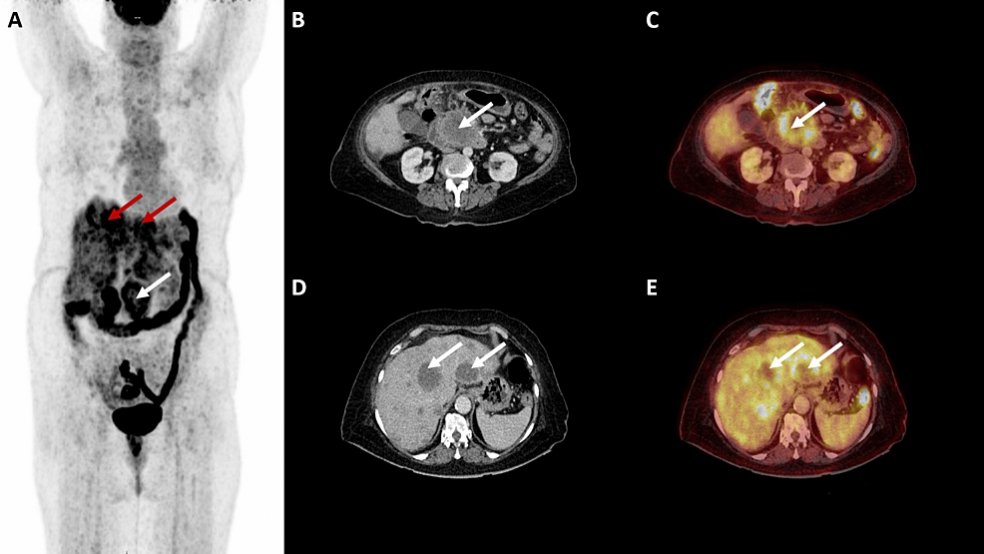
18F-FDG PET/CT images of the patient (A) MIP; (B, D) axial view of CT; (C, E) axial view of 18F-FDG PET/CT fusion. MIP image shows increased FDG uptake in the region of the pancreas (white arrows) and metastatic liver lesions (red arrows). (B, C) Primary neoplastic lesion involving head and uncinate process of pancreas with FDG uptake (white arrows). (D, E) Metastatic liver lesions with FDG uptake (white arrows). MIP: maximal intensity projection; 18F-FDG PET/CT: 18F-flurodeoxyglucose positron emission tomography/computed tomography; FDG: flurodeoxyglucose.

Diagnosis of stage IV pancreatic cancer with liver metastases was established in conjunction with these imaging findings. The patient was explained the stage of the disease and the associated poor prognosis. Subsequently, she was started on palliative chemotherapy with a Gemcitabine and Paclitaxel-based regimen and awaits follow-up (Figures 1A-1E, 2A-2E).

## Discussion

PET/CT is an invaluable tool for staging and response evaluation of most malignant solid tumors in present-day oncology. Many PET agents have been developed and used over time, and 18F-FDG is the most commonly used PET radiotracer worldwide. It has been used extensively over the past few decades and is the agent of choice for imaging of solid tumors like carcinoma lung, breast, lymphomas, etc. However, despite being a standard modality, it is still not a malignancy-specific agent. FDG uptake can be seen in infective and inflammatory conditions [6]. It is also seen as post-treatment inflammatory changes in tumors, at certain times posing a challenge in response evaluation to identify viable residual neoplastic disease. Another limitation is its dependency on blood glucose levels in patients and possible interference with antidiabetic medications and insulin. There has been extensive research in developing malignancy-specific PET agents over time. The development of PET agents like DOTANOC and PSMA is a result of these research initiatives. Radioisotope-labeled FAP inhibitors are today one of the promising PET agents undergoing extensive research. It targets the cancer-associated fibroblasts in tumors and might be an alternative to FDG. Also, it offers the advantage of not being dependent on blood glucose levels in patients. 68Ga-FAPI being produced at the site itself also provides flexibility in performing the PET CT as required and is not dependent on the supply of radiotracer from the cyclotron facility.

In our case study, PET agent FAPI identified the primary disease involving the pancreas with nodal and liver metastases. Compared to FDG PET, the SUVmax values were higher. Also, FAPI was positive in more number of liver lesions and hence more sensitive in identifying the bulk of the disease. Many research publications have emphasized the utility of PET agent FAPI in the detection and staging of various malignancies. Lan et al. compared 68Ga-FAPI and 18F-FDG PET/CT for staging of biliary tract tumors [12]. They reported a higher sensitivity of 68Ga-FAPI PET/CT in detecting primary tumors. 68Ga-FAPI PET/CT resulted in new oncologic findings and upgraded tumor staging or restaging in a subset of patients. In another study, Wang et al. compared 68Ga-FAPI and 18F-FDG PET/CT in the evaluation of advanced lung cancer [13]. In this study, 68Ga-FAPI, in comparison to 18F-FDG, depicted more suspected metastases in lymph nodes, brain, bone, and pleura. Similarly, Liu et al. demonstrated a higher sensitivity of 68 Ga-FAPI PET/CT than 18 F-FDG PET/CT in detecting tumor recurrence, nodal metastases, and distant metastases in gastrointestinal malignancies [14]. As per these studies, PET agent FAPI may be a substitute for 18F-FDG with possible theranostic applications in the future [11, 15]. However, FAPI PET is not completely specific for malignancy. FAPI uptake has been reported in infectious and chronic inflammatory conditions [16]. More data is required to validate the use of FAPI in staging and response evaluation of various malignancies.

## Conclusions

The results of this case discussion demonstrate that PET agent FAPI with selective tumor uptake has the potential for use in the staging and evaluation of malignancies. It not only contributes to the assessment of the burden of metastatic disease but also guides clinical decision-making for relevant therapy. It could potentially be an alternative to FDG PET/CT in uncontrolled diabetic patients, which is a common co-morbidity in cancer patients.
